# Trans catheter embolization of an hepatic artery pseudoaneurysm causing haemobilia: A case report

**DOI:** 10.1016/j.radcr.2026.03.032

**Published:** 2026-04-23

**Authors:** Jihad Karimi, Kawtar Lahlali, Hajar Ouazzani, Ismail Chaouche, Amal Akammar, Nizar El Bouardi, Meriem Haloua, Badreddine Alami, Meryem Boubbou, Mustapha Maaroufi, Moulay Youssef Lamrani Alaoui

**Affiliations:** a Department of Radiology of Specialties, CHU Hassan II, University Sidi Mohamed Ben Abdellah, Fez, Morocco; b Department of Radiology Mother and Child, CHU Hassan II, University Sidi Mohamed Ben Abdellah, Fez, Morocco

**Keywords:** Haemobilia, Hepatic artery pseudoaneurysm, CT angiograph, Transcatheter arterial embolization

## Abstract

Haemobilia, a rare but life-threatening condition, requires prompt diagnosis and intervention due to its high mortality risk. Transcatheter arterial embolization has become the gold-standard treatment, offering both diagnostic confirmation and immediate therapeutic efficacy while minimizing invasiveness. We present a case of haemobilia successfully managed with this approach, underscoring its clinical value.

## Introduction

First characterized by Sandblom in 1948 [1], haemobilia (bleeding into the biliary tree) presents with the complete Quincke's triad of jaundice, abdominal pain, and gastrointestinal bleeding in fewer than 40% of cases [2,3]. This condition most commonly arises from hepatic artery pseudoaneurysms, which develop following laparoscopic interventions [4] or due to anatomical vascular variations [5]. Definitive diagnosis requires contrast-enhanced CT or angiography [6], and emergent transarterial embolization is indicated given the 21%-43% mortality risk associated with pseudoaneurysm rupture [7].

## Case presentation

A 81-year-old woman presented to our institution with abdominal pain, jaundice, and upper gastrointestinal bleeding. Her medical history was significant for stage C acute pancreatitis 2 months prior and laparoscopic cholecystectomy 1 month before admission. Several days postoperatively, she developed febrile jaundice.

The patient developed febrile cholestatic jaundice a few days postoperatively, associated with a progressive deterioration in her general condition. The family was only alerted after the onset of upper gastrointestinal bleeding, consisting of 2 episodes of hematemesis and 1 episode of melena, which occurred 5 days prior to presentation at our institution.

On clinical examination, the patient was icteric and febrile (38.2°C) but hemodynamically stable, with tenderness localized to the right hypochondrium. Laboratory investigations revealed severe anemia: hemoglobin 4.5 g/dL (reference range: 12,5-15,5 g/dL), normal coagulation: prothrombin time 88%, moderate cytolysis: GOT: 188 IU/L (reference range: 0-35 IU/L), marked cholestasis: GGT: 522 IU/L (reference range: 0-55 IU/L), and significant inflammatory response: CRP 85 mg/L (reference range: 0-5 mg/L)

**Fig. 1:**
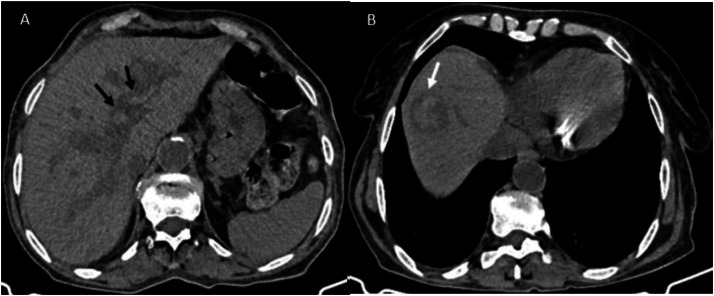
(A) Unenhanced computed tomography shows dilated intrahepatic bile ducts with hyperdense heterogeneous content suggestive of haemobilia (black arrow). (B) It also demonstrates a biloma with hemorrhagic components (white arrow).

**Fig. 2:**
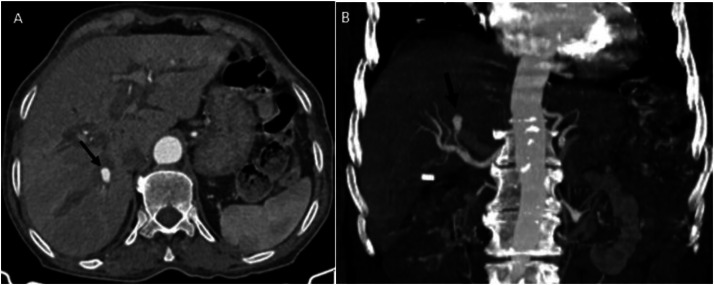
Contrast enhanced computed tomography scan showing an enhancing cavity in segment VII communicating with a sectoral branch of the hepatic artery (black arrow). (A and B).

**Fig. 3:**
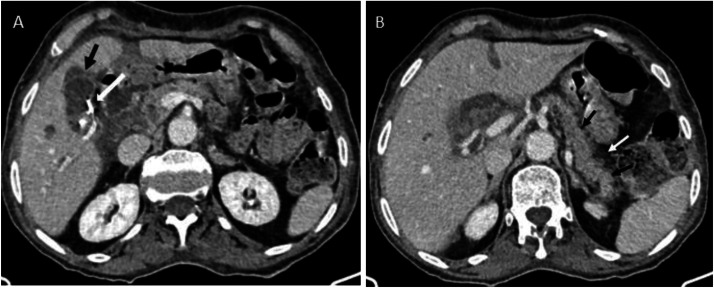
(A) Contrast enhanced computed tomography scan showing a biloma (black arrow) in the glallbladder fossa adjecent to the surgical clip (white arrow). (B) It also demonstrates a peri pancreatic fat infiltration (withe arrow) and pancreatic swelling (black arrow).

Initial abdominal ultrasound demonstrated dilated intrahepatic bile ducts containing echogenic material, a normal-caliber common bile duct, and 2 anechoic collections in the gallbladder fossa. Subsequent multiphase computed tomography revealed key findings: the unenhanced phase showed dilated intrahepatic bile ducts with hyperdense heterogeneous content suggestive of haemobilia (Fig. 1) ; the arterial phase demonstrated a 12 × 7 mm enhancing cavity in segment VII communicating with a sectoral branch of the hepatic artery; (Fig. 2) and the venous phase confirmed intrahepatic bilomas and in the glallbladder fossa one with hemorrhagic components along with residual features of stage C pancreatitis (Fig. 3).

These imaging findings established the diagnosis of a segment VII hepatic artery pseudoaneurysm with secondary haemobilia.

The patient was admitted to the medical intensive care unit, where she was closely monitored and received initial resuscitative management. Volume resuscitation was initiated with colloid fluids, followed by transfusion of 2 units of packed red blood cells. Broad-spectrum antibiotic therapy with piperacillin–tazobactam was also initiated.

**Fig. 4:**
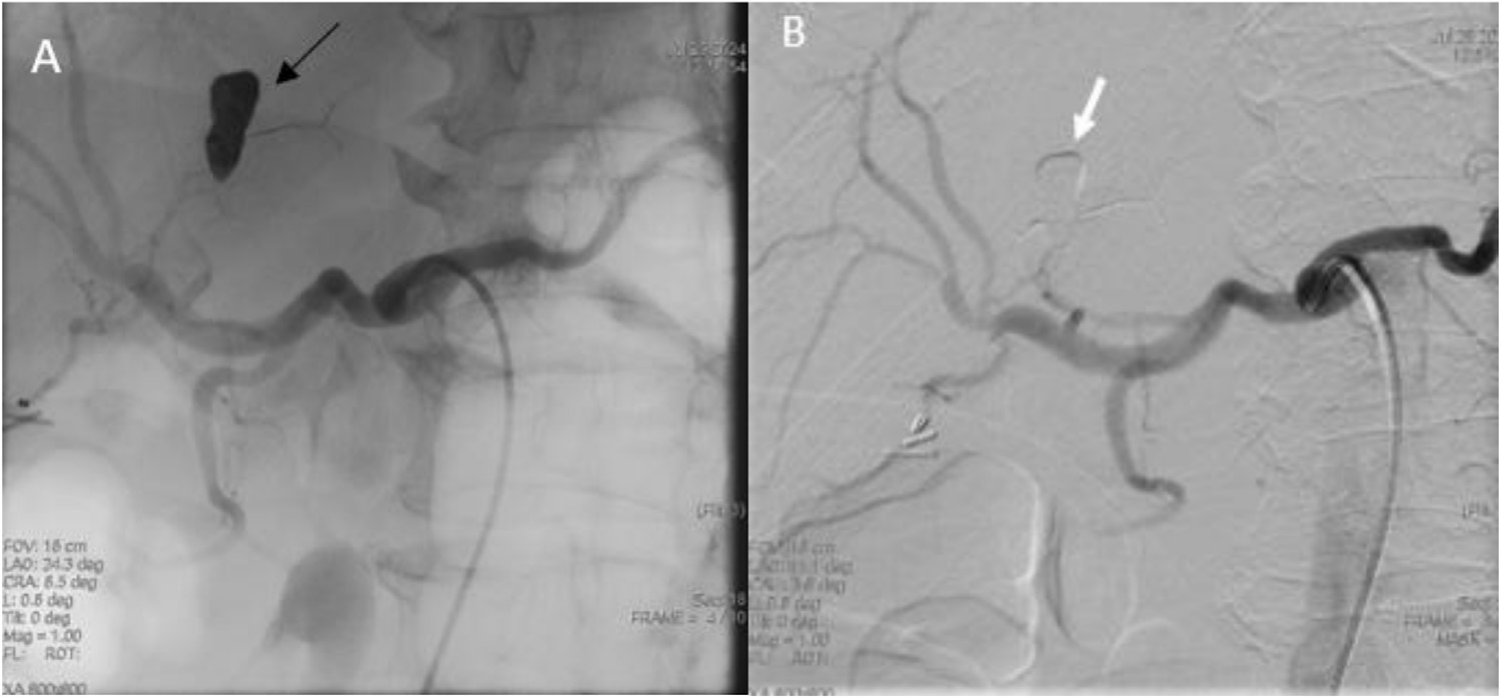
(A) Native selective hepatic artery angiography after hyper selective embolization with Onyx™ (black arrow). (B) Substracted image with embolization of the feeding artrey (white arrow).

Definitive treatment was achieved the following day through transcatheter arterial embolization. The pseudoaneurysm was precisely localized by digital subtraction angiography and effectively embolized with 1 ml of Onyx™ (ethylene-vinyl alcohol copolymer) injected through a detachable tip. Post-procedural imaging confirmed complete exclusion of the pseudoaneurysm with preservation of hepatic parenchymal perfusion (Fig. 4).

**Fig. 5:**
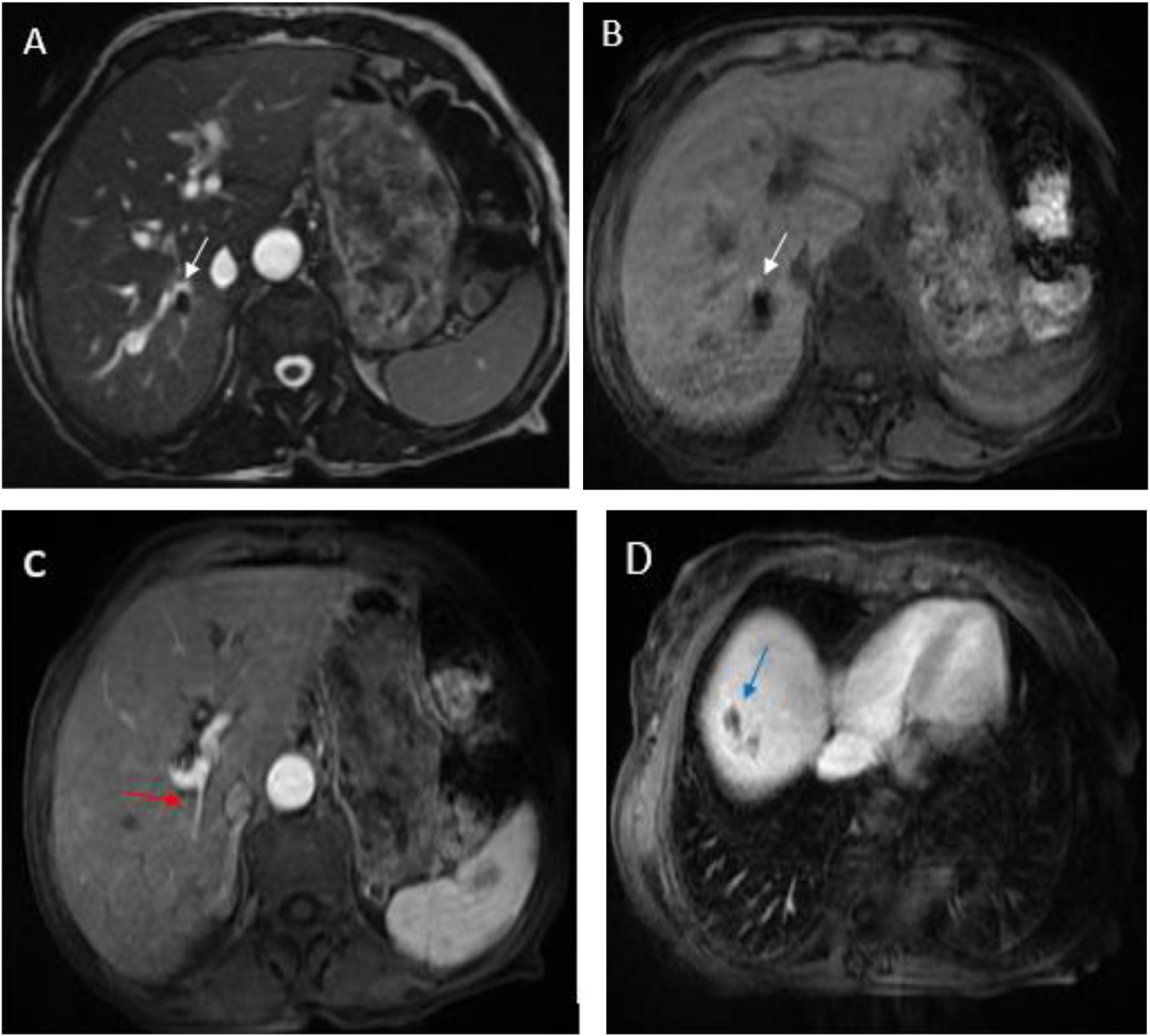
FIESTA and T1-weighted axial sequences, demonstrating the embolization material as T1 and T2 hypointense signal (white arrow) (A and B) with a regular appearance of the hepatic artery wall (red arrow) (C). There is also a significant reduction in the hepatic fluid collection, corresponding to a biloma (blue arrow)(D)**.**

Following the embolization procedure, our patient demonstrated significant clinical improvement with stabilization of hemoglobin levels. In our case, the patient was monitored with an MRI 1 month later, which demonstrated a significant regression of the biloma in segment VII. The imaging also showed the visualization of embolization material projected over the divisional branches of the hepatic artery and the absence of evidence of the pseudoaneurysm (Fig. 5).

## Discussion

Biliary hemorrhage, first introduced by Sandblom under the term haemobilia, refers to bleeding that occurs within the biliary tree, with blood passing through the papilla of Vater into the gastrointestinal lumen [1].

Fewer than 40% of haemobilia patients exhibit the classic symptoms of Quincke’s triad (jaundice, abdominal pain, and GI bleeding) [2]. Occasionally, an abdominal mass may be present [3]. In our case, the patient had the complete Quincke triad.

While the underlying mechanisms are not fully elucidated, factors such as surgical vascular injury, stray electrical current transmission via adjacent clips, and vascular damage from clip migration may contribute to the development of hepatic artery pseudoaneurysms [3].

**Fig. 6:**
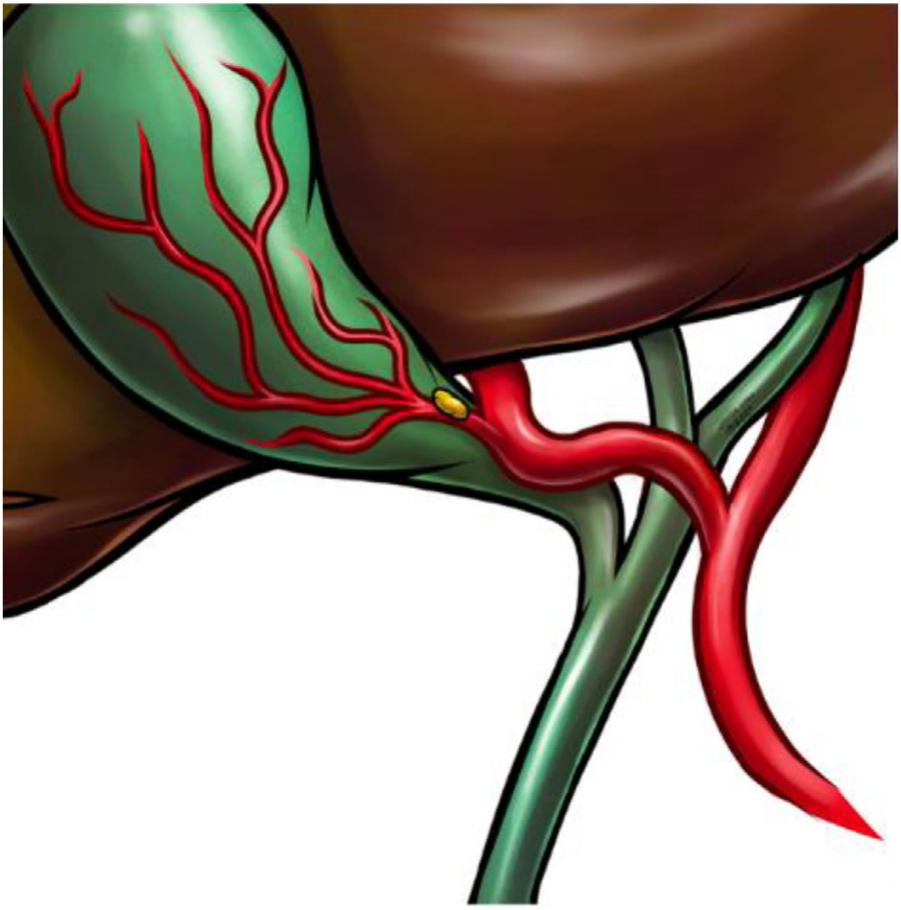
Diagram representing Moynihan's hump or caterpillar hump, with a single loop and the cystic artery short in its convexity [8].

Another possible explanation is anatomical variations, particularly those involving the right hepatic artery (RHA) forming a "caterpillar hump" configuration [5] (Fig. 6).

In our case, the intrahepatic location of the pseudoaneurysm makes its mechanism enigmatic. We considered a mycotic origin, even though the clinical presentation does not support it. Intraoperative traction on the hepatic hilum remains a probable etiology, whereas a direct surgical vascular injury is unlikely due to the pseudoaneurysm's specific location.

Evidence from peer-reviewed studies reveals the paradoxical finding that minimally invasive laparoscopic techniques correlate with elevated hepatic artery pseudoaneuvrysm (HAP) rates compared to traditional open surgeries [4].

Clinical observations demonstrate that HAPs frequently manifest weeks to months postoperatively, most commonly presenting as varying degrees of gastrointestinal hemorrhage due to haemobilia, often resulting in diagnostic challenges and treatment delays [9]. While symptom onset occurred in the early postoperative period, our patient deferred medical attention for 4 weeks, presenting only after experiencing progressive functional decline.

Pseudoaneurysm diagnosis represents a critical surgical emergency requiring urgent intervention, as rupture carries a significant mortality risk (21%-43%) due to potentially fatal blood loss [7].

The diagnosis of a pseudoaneurysm (PSA) is established through a combination of clinical evaluation and imaging studies. A thorough patient history, particularly regarding previous surgical interventions, represents the first diagnostic step. Imaging modalities include upper gastrointestinal endoscopy, ultrasonography (with Doppler capabilities), contrast-enhanced CT scans, and angiography. While ultrasound can detect aneurysmal structures or hemorrhagic fluid, its diagnostic accuracy depends on the lesion size and operator experience. Currently, multidetector CT scans with 3D reconstruction capabilities have become the preferred initial imaging modality due to their ability to provide detailed vascular anatomy and volume assessment. Angiography remains the definitive diagnostic method and also offers therapeutic potential. Characteristic CT findings suggestive of haemobilia include intra-abdominal hematomas (particularly in the gallbladder fossa), hyperdense blood clots within the bile ducts, biliary dilatation, right hepatic artery pseudoaneurysms, contrast extravasation, enhanced bile duct walls, and right hepatic lobe hypoperfusion. These imaging features, when correlated with clinical presentation, enable accurate PSA diagnosis and guide appropriate management [6]. In our patient, the diagnosis was established through comprehensive evaluation of cross-sectional imaging findings in conjunction with the patient's clinical presentation and prior surgical history.

The surgical management of hepatic artery aneurysms (HAAs) involves various techniques, such as ligation, complete aneurysm removal (with or without arterial reconstruction using venous or synthetic grafts), and arterial bypass. For intrahepatic aneurysms, transarterial embolization is the treatment of choice. It has also been explored as an initial option for high-risk patients with extrahepatic aneurysms. However, embolization carries a failure rate of approximately 10%-15%, with procedural complications occurring in 14%-25% of cases [10].

Potential complications include rupture during coil deployment, thrombotic extension into the right hepatic artery (RHA), hepatobiliary necrosis, hemorrhage, abscess formation, and ischemic strictures of the common bile duct [11].

According to a recent study, the post-embolization syndrome occurrence is linked to both the patient's age and the duration between laparoscopic cholecystectomy (LC) and transarterial embolization [6].

Transarterial embolization involves sealing the aneurysm sac or its feeding vessel using various embolic materials such as gelatin sponge, coils, N-butyl cyanoacrylate (NBCA), or thrombin. To minimize collateral refilling of the PSA, optimal technique involves occluding the vessel both proximal and distal to the aneurysm. However, while coils promote thrombosis, they may fail to achieve complete vessel occlusion in patients with severe coagulopathy, potentially leading to persistent bleeding. For smaller PSAs, liquid embolic agents like glue may be preferable, as they adapt to the aneurysm's shape [12]. Coil deployment can also be technically challenging in cases with very small PSAs. In certain scenarios, a combination of embolic agents may be employed to enhance treatment efficacy [13].

NBCA is an adhesive liquid embolic that rapidly polymerizes upon contact with blood, allowing prompt and effective occlusion of the target vessel. Its polymerization time can be modulated by dilution with ethiodized oil, enabling controlled delivery under fluoroscopic guidance. However, the rapid polymerization of NBCA requires precise injection technique and carries a potential risk of non-target embolization in cases of reflux or distal migration. In contrast, Onyx is a non-adhesive liquid embolic agent that allows slower and more controlled injection due to its cohesive properties. This characteristic facilitates progressive penetration into the pseudoaneurysm sac and feeding vessels while reducing the risk of catheter adhesion. Nevertheless, Onyx typically requires longer injection times and specific preparation prior to use. Consequently, the choice between NBCA and Onyx often depends on vascular anatomy, operator experience, and the availability of embolic materials, particularly in emergency settings [14].

In our case, the use of Onyx was preferred due to its ease of use, the convenience of administration, as well as the operator’s preference.

Recent studies have documented successful treatment of PSAs through direct thrombin injection into the hepatic artery aneurysm. While this approach shows promise, it carries a risk of non-selective embolization. To enhance treatment safety, researchers recommend administering thrombin in small, controlled doses under continuous ultrasound and Doppler monitoring [12].

Based on clinical experience, the authors propose at least one follow-up imaging evaluation approximately 6 months post-intervention, using abdominal CT scans with 3-mm slice thickness as the preferred imaging modality. If this follow-up examination shows no evidence of recurrent hepatic pseudoaneurysm, then no further studies need to be obtained [15].

## Conclusion

Haemobilia in the context of a cholecystectomy should raise suspicion for a pseudoaneurysm of one of the hepatic artery branches. Esophagogastroduodenoscopy (EGD) is performed as the first-line examination to rule out another cause of the gastrointestinal hemorrhage, and abdominal CT angiography (CTA) is the key test for diagnosing the pseudoaneurysm. Highly selective catheterization of the pseudoaneurysm yields good results with low morbidity.

## Ethics approval and consent to participate

Ethical approval was obtained in accordance with institutional guidelines. Written informed consent to participate was obtained from all patients included in this study.

## Availability of data and material

All data generated or analyzed during this study are included in this published article. Additional information is available from the corresponding author upon reasonable request.

## Author’s contribution

JK Is the corresponding author, she participated in the organization and writing of the article. Professor MYA supervised the working and validated the figures. Professor and chief of department of radiology MB and MM red and allowed the article for publication. All authors read and approved the final manuscript.

## Patient consent

Written informed consent was obtained from the patient for publication of this case report and accompanying images. The patient has consented to the submission of this case report to the journal.
